# Postbiotic supplementation promotes gut barrier integrity and immune balance in cats via microbiota modulation

**DOI:** 10.3389/fmicb.2025.1692845

**Published:** 2025-10-24

**Authors:** Weiwei Wang, Chengyi Miao, Qianqian Chen, Longjiao Wang, Xiaohan Chang, Lili Zhao, Wenjing Ma, Shanyu Lei, Mengyao Ma, Yuqiang Zhang, Shuxing Chen, Lishui Chen, Ran Wang, Shaoyang Ge, Wei Xiong

**Affiliations:** ^1^Food Laboratory of Zhongyuan, College of Food Science and Technology, Henan University of Technology, Zhengzhou, Henan, China; ^2^Henan Zhiyuan Henuo Technology Co., Ltd., Luohe, China; ^3^Key Laboratory of Precision Nutrition and Food Quality, Department of Nutrition and Health, China Agricultural University, Beijing, China

**Keywords:** postbiotics, gut microbiota, feline health, immune modulation, intestinal barrier

## Abstract

**Introduction:**

Gastrointestinal disorders are common in cats and are closely linked to the gut microbiota, which supports immune function, metabolic balance, and barrier integrity. Although probiotics are beneficial, they can be unstable in pet foods. Postbiotics offer a safer and more stable alternative.

**Methods:**

A postbiotic derived from *Bifidobacterium animalis* subsp. *lactis* was prepared by heat inactivation (95°C, 30 min), followed by centrifugation and freeze-drying. Eighteen healthy domestic cats were randomly assigned to a control group, a low-dose postbiotic group, or a high-dose postbiotic group for 28 days. Assessments included physical condition, immune status, gut barrier function, microbiota composition, and fecal short-chain fatty acids (SCFAs).

**Results:**

Postbiotic supplementation improved fur condition and enhanced gut barrier integrity, as indicated by reduced levels of permeability markers such as diamine oxidase (DAO) and lipopolysaccharide (LPS). It also promoted immune function by increasing serum immunoglobulin G and anti-inflammatory cytokines IL-10 and IL-4. In addition, the intervention enriched beneficial bacteria such as *Bifidobacterium* spp. and elevated fecal concentrations of key SCFAs, including acetic, propionic, and butyric acids.

**Discussion:**

These findings support the use of postbiotics as a safe and effective nutritional approach to help maintain gastrointestinal and immune health in cats.

## 1 Introduction

The gut microbiota plays a vital role in the overall health of companion animals, influencing digestion, immune regulation, and metabolic homeostasis. Recent studies have shown that the composition of the intestinal microbiome is closely linked to various physiological functions, including nutrient absorption, gut barrier integrity, and host immunity (Hosseini et al., 2024). In pets, an imbalanced gut microbiota, often referred to as dysbiosis, has been associated with digestive disorders, inflammatory diseases, and metabolic disturbances (Kayser et al., 2024). Probiotics, which are live beneficial microorganisms, have been widely used to support gut health in pets by restoring microbial balance and promoting the production of beneficial metabolites (Mosca et al., 2022). However, the efficacy of probiotics is often compromised by factors such as strain viability, stability during storage, and survival in the gastrointestinal tract (Sabahi et al., 2023). Additionally, probiotics may pose risks for immunocompromised animals, as live bacterial strains have the potential to translocate across the gut barrier and cause infections (Saettone et al., 2020). These limitations have driven interest in alternative approaches, particularly postbiotics, which provide similar benefits without the drawbacks of live microorganisms.

Postbiotics are bioactive compounds derived from inactivated beneficial microorganisms or their metabolites, including short-chain fatty acids (SCFAs), peptides, exopolysaccharides, and enzymes (Vinderola et al., 2022). Extensive research has confirmed their diverse beneficial properties, notably anti-inflammatory, immunomodulatory, gut barrier-enhancing, and antimicrobial effects. For instance, certain postbiotic formulations effectively decrease pro-inflammatory cytokines such as TNF-*α* and IL-6, while concurrently increasing anti-inflammatory cytokines like IL-10 (Feng et al., 2024). Additionally, postbiotics strengthen intestinal barrier integrity through the upregulation of tight junction proteins, thereby mitigating gut permeability and associated endotoxemia (Li et al., 2024a). Moreover, postbiotics demonstrate notable antimicrobial activity, inhibiting pathogenic bacterial proliferation without promoting antimicrobial resistance, which is an increasing concern with traditional antimicrobial agents (Saettone et al., 2020). In contrast to probiotics, postbiotics circumvent the necessity for microbial viability and gastrointestinal tract survivability, thus providing greater stability, consistency, and safety in functional pet food applications (Mosca et al., 2022; Zhao et al., 2024).

Recent scientific literature emphasizes the expanding integration of postbiotics into functional pet food formulations owing to their beneficial roles in digestive health, immune modulation, and metabolic enhancement (Sabahi et al., 2023). Specifically, postbiotics augment SCFA synthesis, which supports microbiome equilibrium and serves as a vital energy substrate for intestinal epithelial cells (Hosseini et al., 2024). Furthermore, evidence indicates their efficacy in managing gastrointestinal disorders such as diarrhea, irritable bowel syndrome, and inflammatory bowel disease, primarily through their capacity to modulate gut microbiota composition and prevent dysbiosis (Kayser et al., 2024; Feng et al., 2024). Long-term dietary inclusion of postbiotics in pet nutrition has also been associated with improved dermatological conditions, enhanced immunological resilience, and overall health and wellness, underscoring their potential as a valuable component of next-generation pet nutrition strategies (Zhao et al., 2024).

To our knowledge, the impact of dietary postbiotics on feline health remains underexplored, despite the unique physiological and microbiota characteristics of cats. In this study, we aim to systematically investigate the effects of postbiotic supplementation on feline gastrointestinal and immune health by assessing key physiological parameters, blood profiles, immune markers, inflammatory responses, microbial composition, and SCFA concentrations. These findings are expected to provide novel insights into species-specific nutritional strategies and to highlight the potential of postbiotics as a safe and effective approach for promoting gut health in cats.

## 2 Materials and methods

### 2.1 Animals and experimental treatments

The animal study protocol was reviewed and approved by the Institutional Animal Care and Use Committee of China Agricultural University (Approval No.: AW42405202-1-01). All procedures involving animals strictly adhered to international guidelines for animal research and the Regulations for the Administration of Affairs Concerning Experimental Animals in China, with maximal efforts made to ensure animal welfare and minimize discomfort.

Eighteen healthy Chinese domestic shorthair cats (orange tabby coat, male: female = 1:1), aged approximately 1 year (range: 11–12 months), were randomly allocated into three groups (*n* = 6 per group): control group (CG), low-dose postbiotic group (LDP, 250 mg/kg body weight), and high-dose postbiotic group (HDP, 500 mg/kg body weight). Postbiotics were obtained from whole inactivated cells of *Bifidobacterium animalis* subsp. *lactis* ZYhyy-003 (CGMCC No. 31553) (2 × 10^10^ CFU/mL) by subjecting the culture to heat inactivation at 95 °C for 30 min after fermentation, followed by centrifugation and freeze-drying. The effectiveness of the heat inactivation was confirmed by plating the treated culture on MRS agar and verifying the absence of bacterial growth. The preparation was carried out by Beijing HeyiYuan Biotechnology Co., Ltd. Prior to the experiment, all cats were immunized with a commercial inactivated feline vaccine (Miao San Duo, Zoetis, China), which contains multiple inactivated antigens and is safe without causing clinical symptoms, dewormed, and not exposed to antibiotics or microbiota-altering treatments for at least 4 weeks. Cats were individually housed under a 12-h light–dark cycle, temperatures maintained between 20 and 25 °C, and relative humidity at 45–65%. Cages were cleaned daily, and feces removed twice daily. All cats were fed a nutritionally complete diet (AAFCO/NRC standards) twice daily (35 g per meal), with ad libitum access to fresh water.

After a seven-day acclimatization, LDP and HDP groups received their respective daily postbiotics supplements based on body weight, mixed thoroughly with their diet for 28 consecutive days. Body weight, general physical condition, fur quality, and fecal characteristics were recorded on days 0, 7, 14, 21, and 28 to evaluate the effects of postbiotic supplementation.

### 2.2 Sample collection

On day 28, approximately 1.5 mL of blood was collected from the cephalic vein of each cat using a sterile syringe. From each blood sample, 0.5 mL was immediately transferred into a vacutainer containing heparin sodium for hematological analysis. The remaining blood was centrifuged at 3000 × g for 15 min at 4 °C, after which plasma was carefully collected and stored at −20 °C until further biochemical analyses. Fresh fecal samples were collected directly from sterile paper placed within each cat’s litter box. Using sterilized forceps, approximately 6 g of feces per cat was carefully transferred into sterile 50 mL tubes, taking precautions to avoid contamination. Fecal samples were immediately frozen and preserved at −80 °C for microbiota and short-chain fatty acid analyses. In addition, fur samples from the dorsal region of each cat were obtained using sterilized scissors. Hair samples were uniformly collected from identical locations on each cat’s back, placed in sealed bags, and stored at room temperature until scanning electron microscopy (SEM) analysis. For SEM evaluation, multiple hairs were collected from each individual to account for intra-individual variation. Representative images were then selected after consistent evaluation across samples, ensuring that the *n* = 6 per group reflects independent biological replicates (cats).

### 2.3 Fur condition and fecal score

The fecal quality scoring was conducted using previously established methods by Carciofi et al. (2008). Fur quality assessment was performed visually by an experienced investigator, applying a three-point scale: score 3 indicated excellent fur condition (clean and smooth appearance), score 2 represented intermediate condition (partially clean and slightly disheveled), and score 1 corresponded to poor condition (rough, disheveled appearance). All evaluations were performed by trained veterinarians who were blinded to the treatment groups, and assessments were conducted consistently on days 0, 7, 14, 21, and 28 throughout the experimental period.

### 2.4 Hematological and biochemical analysis

Blood and serum samples were collected and analyzed for comprehensive hematological, immune, inflammatory, and intestinal barrier integrity parameters. A complete blood count (CBC) was performed using an automated hematology analyzer (Contec, Qinhuangdao, Hebei, China) to determine routine hematological indices. Serum concentrations of intestinal barrier integrity markers, including diamine oxidase (DAO), D-lactate (D-LA), and lipopolysaccharide (LPS), were quantitatively measured utilizing enzyme-linked immunosorbent assay (ELISA) kits according to the manufacturer’s guidelines (Shanghai Enzyme-linked Biotechnology Co., Ltd., Shanghai, China). Similarly, inflammatory cytokines comprising tumor necrosis factor-alpha (TNF-*α*), interferon-gamma (IFN-*γ*), C-reactive protein (CRP), interleukin-1 beta (IL-1β), interleukin-4 (IL-4), interleukin-6 (IL-6), and interleukin-10 (IL-10) were assessed by ELISA using commercial assay kits obtained from the same supplier. Additionally, Serum immunoglobulin profiles (IgA, IgG, IgM) were measured using a turbidimetric immunoassay, following the manufacturer’s protocol (Shanghai Enzyme-linked Biotechnology Co., Ltd., China). All assays were performed in duplicate, and mean values were used for statistical analysis to ensure accuracy and reproducibility.

### 2.5 16S rRNA gene sequencing and microbial bioinformatics analysis

Total microbial DNA was isolated from these samples using the E. Z. N. A.® Soil DNA Kit (Omega Biotek, Norcross, GA, USA), strictly adhering to the manufacturer’s protocol. The integrity and quality of the extracted DNA were verified by electrophoresis on 1% agarose gels, and DNA concentration and purity were quantified with a NanoDrop™ 2000 spectrophotometer (Thermo Scientific, Waltham, MA, USA). Acceptable purity levels were indicated by A260/280 ratios exceeding 1.8 and A260/230 ratios above 2.0. PCR amplification targeting the V3–V4 hypervariable regions of the bacterial 16S rRNA gene was performed using primer pairs 338F (5′-ACTCCTACGGGAGGCAGCAG-3′) and 806R (5′-GGACTACHVGGGTWTCTAAT-3′). Amplification reactions were conducted in triplicate to enhance accuracy and reliability, and PCR products were pooled, purified using the AxyPrep DNA Gel Extraction Kit (Axygen Biosciences, Union City, CA, USA), and quantified with a Quantus™ Fluorometer (Promega Corporation, Madison, WI, USA).

The purified amplicons were used for library preparation with the NEXTflex™ Rapid DNA-Seq Kit (Bioo Scientific, Austin, TX, USA) and sequenced on an Illumina NovaSeq 6,000 platform (Illumina, San Diego, CA, USA) with 250 bp paired-end reads. Raw sequencing data were demultiplexed and quality-filtered using fastp (v0.20.0), and paired-end reads were merged with FLASH (v1.2.11). High-quality sequences were clustered into operational taxonomic units (OTUs) at a 97% similarity threshold using UPARSE (v11.0.667), and chimeric sequences were removed during this process. Taxonomic classification of representative sequences was performed with the RDP Classifier against the SILVA 138.2 database at a confidence threshold of 70%. Microbial *α*-diversity indices (including Chao1 and Shannon) and *β*-diversity metrics (Bray–Curtis dissimilarity, principal coordinate analysis (PCoA)) were calculated using QIIME 2. Functional prediction of microbial communities was carried out using PICRUSt2. Statistical analyses, including Kruskal–Wallis tests and Spearman correlation heatmaps linking bacterial communities with host biomarkers, were performed in R software (v4.2.0). For correlation analyses, *p*-values were adjusted for multiple comparisons using the Benjamini–Hochberg false discovery rate (FDR) method, and significance was determined at *p* < 0.05 after correction.

### 2.6 Short-chain fatty acids

SCFAs were determined using gas chromatography. Briefly, approximately 50 mg of sample was homogenized with diluted sulfuric acid, diethyl ether, and an internal standard, followed by centrifugation at 12,000 × g for 10 min at 4 °C. The resulting supernatant was filtered (0.22 μm) and analyzed by GC equipped with an Agilent DB-WAX UI column (30 m × 0.25 mm × 0.25 μm) and a flame ionization detector. The oven temperature program started at 60 °C, held for 4 min, increased to 180 °C at 6 °C/min, then further increased to 200 °C at 20 °C/min and maintained for 10 min. Injector and detector temperatures were set at 250 °C, and nitrogen was employed as the carrier gas at a flow rate of 1 mL/min. Calibration standards were prepared from stock solutions of acetic, propionic, butyric, valeric and isovaleric acids for quantitative analysis.

### 2.7 Statistical analyses

Experimental data were statistically analyzed using IBM SPSS Statistics software (version 24.0; IBM Corp., Armonk, NY, USA). Normality of data distribution was assessed using the Shapiro–Wilk test. For variables meeting normality assumptions, one-way analysis of variance (ANOVA) was performed, followed by Tukey’s post-hoc test for multiple comparisons. For datasets not conforming to normal distributions, including most microbiota relative abundance data and certain cytokine measurements, non-parametric tests such as the Kruskal–Wallis H test were applied. A *p*-value less than 0.05 was considered statistically significant. Graphs were generated using GraphPad Prism software (version 10.1.2; GraphPad Software, San Diego, CA, USA).

## 3 Results

### 3.1 Physical condition and growth performance

**Figure 1:**
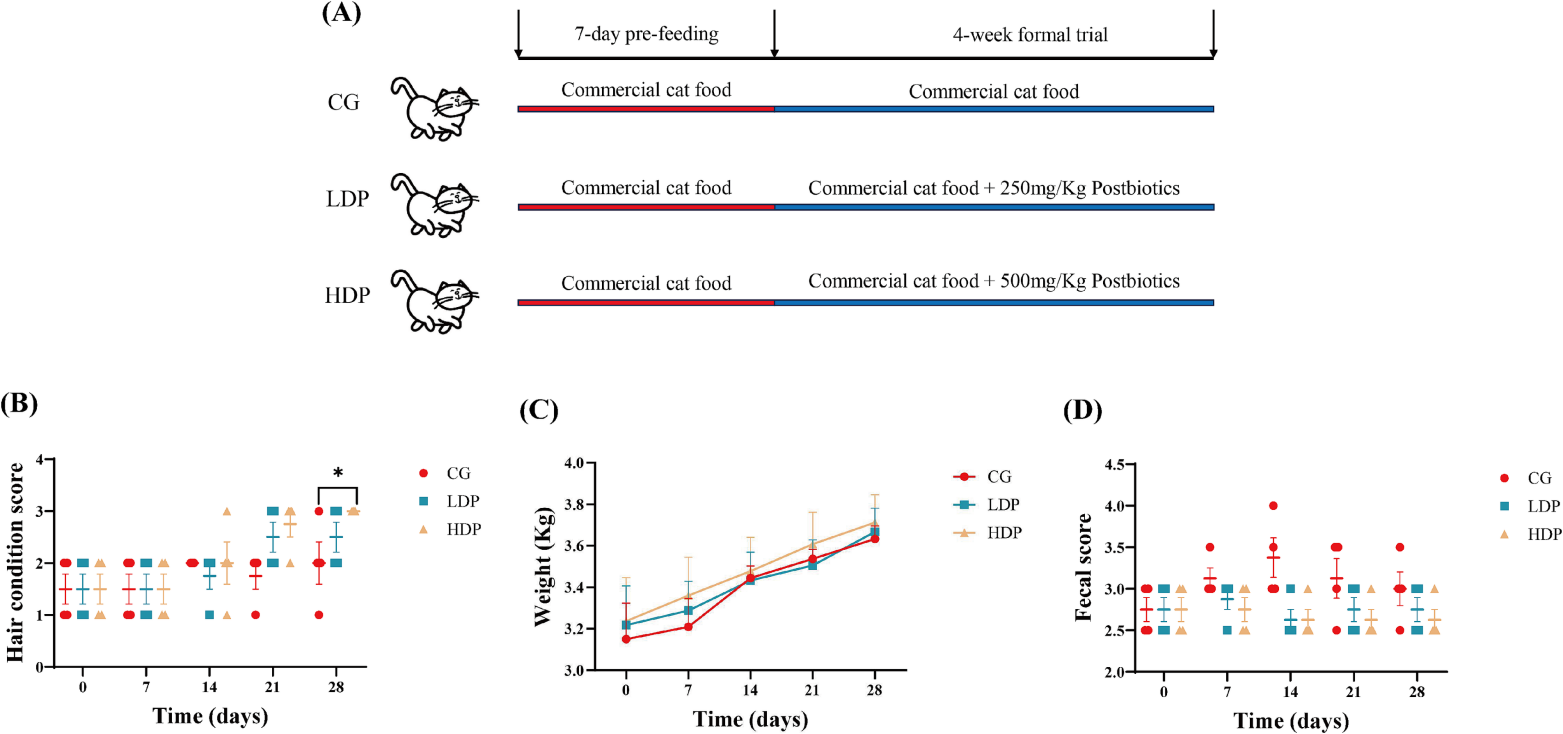
Effects of postbiotics on growth performance in cats. **(A)** Design of the trial, **(B)** Fur condition score, **(C)** Body weight, and **(D)** Fecal score. CG: Control Group, LDP: Low-Dose Postbiotics, HDP: High-Dose Postbiotics. The values are expressed as means ± SEM, *n* = 6. Significant differences are indicated as * *p* ≤ 0.05.

The physical condition of cats, including hair quality, body weight, and fecal scores, is summarized in Figure 1. Fur condition scores increased over time in all groups, with the HDP groups showing higher scores than the control group, particularly at day 28 (Figure 1B). Body weight increased steadily in all groups, with no significant differences among them (Figure 1C). Fecal scores showed little variation between groups, although scores in the LDP and HDP groups clustered around 2.5–3.0 (Figure 1D).

### 3.2 Hair structure analysis

**Figure 2:**
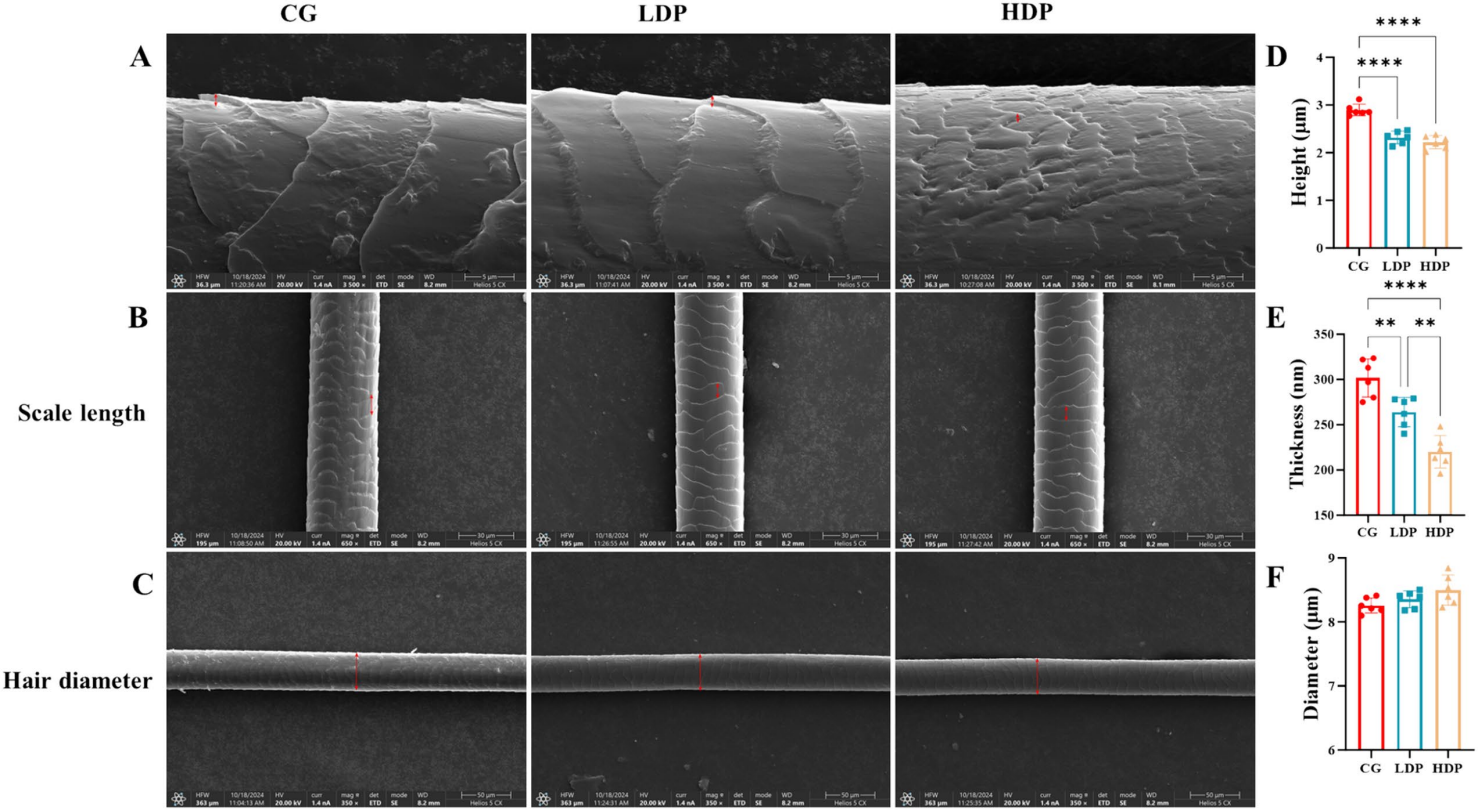
Effects of postbiotics on cat hair structural characteristics. **(A)** SEM images showing hair scale thickness in CG (Control Group), LDP (Low-Dose Postbiotics), and HDP (High-Dose Postbiotics), Scale bars = 10 μm. **(B)** SEM images showing hair scale length in CG, LDP, and HDP, Scale bars = 30 μm. **(C)** SEM images showing hair diameter in CG, LDP, and HDP, Scale bars = 50 μm. **(D–F)** Quantitative comparisons of hair scale thickness, length, and diameter among CG, LDP, and HDP, respectively. Data are expressed as means ± SEM (*n* = 6). Statistical significance was analyzed using one-way ANOVA followed by Tukey’s post-hoc test. Significant differences are indicated as * *p* ≤ 0.01**, *p* ≤ 0.01 and *** *p* ≤ 0.001.

Scanning electron microscopy analysis revealed distinct structural variations in cat hair among the experimental groups, particularly in terms of hair scale thickness and hair scale length, as illustrated in Figures 2A–C. The CG group exhibited a rougher hair surface with more pronounced cracks and scale separation, whereas the LDP and HDP groups displayed relatively smoother surfaces with fewer structural irregularities.

Quantitative analysis further confirmed that the mean hair scale thickness was 301.75 nm in the CG group, 264.00 nm in the LDP group, and 220.00 nm in the HDP group (Figure 2D). A statistically significant reduction in thickness was observed in the HDP group compared to both the CG and LDP groups, suggesting a dose-dependent effect of postbiotic treatment. As depicted in Figure 2E, the hair scale length of cat hair in the CG, LDP, and HDP groups was 2.90 μm, 2.32 μm, and 2.22 μm, respectively. Postbiotic treatment resulted in a reduction in cuticle length, although no significant difference was observed between the LDP and HDP groups. Furthermore, as shown in Figure 2F, the hair diameter measured 8.26 μm in the CG group, 8.36 μm in the LDP group, and 8.50 μm in the HDP group, with no statistically significant differences detected among the three groups.

### 3.3 Complete blood count

The CBC parameters of the cats are shown in Table 1. The CBC indices of the CG, LDP, and HDP groups all fell within the normal healthy range, and no significant differences were observed among the three groups.

**Table 1:** The influence of postbiotics on cat complete blood count (means ± SEM, *n* = 6 per group).

Item	CG	LDP	HDP	Normal reference range	*p-*value
Total white blood cells (10^9^/L)	19.38 ± 1.56	15.50 ± 2.07	14.63 ± 0.46	5.5–19.5	0.09
Lymphocyte ratio (%)	34.47 ± 1.60	41.08 ± 1.71	44.77 ± 2.51	12.0–45.0	0.89
Intermediate cell ratio (%)	7.85 ± 0.35	7.78 ± 0.67	7.55 ± 0.78	2.0–9.0	0.94
Granulocyte ratio (%)	41.65 ± 2.76	41.15 ± 2.80	38.80 ± 4.16	35.0–85.0	0.85
Lymphocytes (10^9^/L)	6.68 ± 1.03	6.48 ± 0.47	6.55 ± 0.61	0.8–7.0	0.98
Intermediate cells (10^9^/L)	1.53 ± 0.15	1.70 ± 0.30	1.43 ± 0.17	0.1–1.9	0.69
Granulocytes (10^9^/L)	6.18 ± 1.20	5.83 ± 0.57	4.90 ± 0.91	2.1–15.0	0.67
Total red blood cells (10^12^/L)	9.14 ± 0.39	8.27 ± 0.22	9.01 ± 0.47	4.60–10.00	0.29
Hemoglobin (g/L)	139.75 ± 6.91	122.75 ± 1.75	139.75 ± 12.78	93–153	0.33
Hematocrit (%)	38.85 ± 1.38	37.50 ± 0.53	41.25 ± 3.05	28.0–49.0	0.44
Mean corpuscular volume (fL)	42.63 ± 0.62	45.53 ± 1.18	45.68 ± 1.03	39.0–52.0	0.10
Hemoglobin content (pg)	15.23 ± 0.32	14.83 ± 0.23	15.38 ± 0.61	13.0–21.0	0.67
Hemoglobin concentration (g/L)	358.75 ± 5.28	327.25 ± 4.52	337.00 ± 6.06	300–380	0.06
Red cell distribution width SD (fL)	47.35 ± 6.99	49.48 ± 5.61	52.90 ± 6.74	47.0–62.7	0.85
Red cell distribution width CV (%)	17.15 ± 0.45	16.08 ± 0.81	14.95 ± 0.32	14.0–18.0	0.08
Total platelet count (10^9^/L)	331.50 ± 77.02	204.50 ± 53.15	328.25 ± 127.91	100–514	0.58
Mean platelet volume (fL)	9.50 ± 0.61	10.68 ± 1.46	10.65 ± 0.17	5.0–11.8	0.62
Platelet distribution width (%)	9.48 ± 0.43	9.78 ± 1.85	11.03 ± 1.30	0.1–30.0	0.70
Plateletcrit (%)	0.32 ± 0.09	0.24 ± 0.09	0.42 ± 0.21	0.01–9.99	0.69
Platelet–larger cell ratio (%)	17.53 ± 3.23	15.83 ± 5.89	23.85 ± 2.63	0.1–99.9	0.42

### 3.4 Gut barrier function

**Figure 3:**
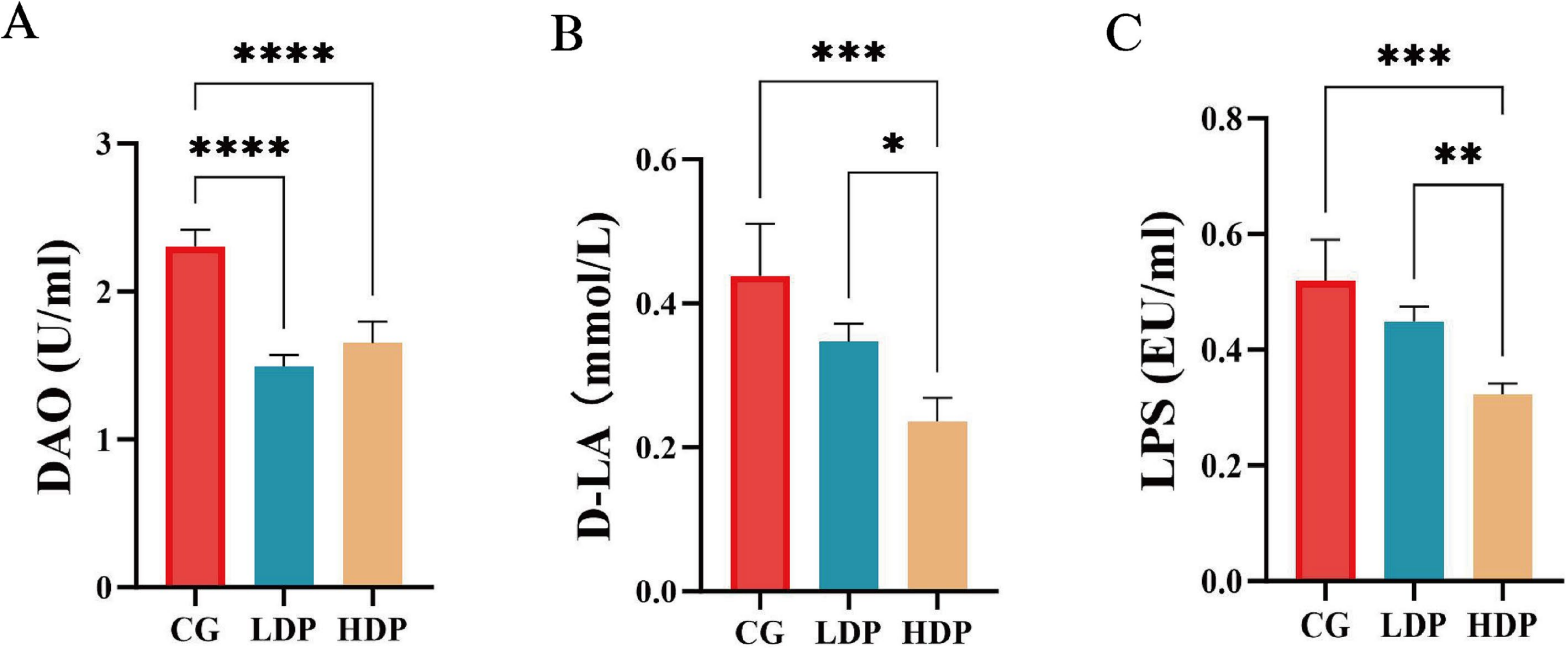
Effects of postbiotics on plasma intestinal barrier function parameters in cats. **(A)** DAO, diamine oxidase, **(B)** D-LA, D-lactate and **(C)** LPS, Lipopolysaccharide. Data are expressed as means ± SEM (*n* = 6). Statistical significance was analyzed using one-way ANOVA followed by Tukey’s post-hoc test. Significant differences are indicated as * *p* ≤ 0.05**, *p* ≤ 0.01, *** *p* ≤ 0.001 and **** *p* ≤ 0.0001.

Figure 3 illustrates the measured concentrations of DAO, D-LA, and LPS, key indicators of gut barrier integrity. The DAO levels in the CG, LDP, and HDP groups were 2.31 U/mL, 1.49 U/mL, and 1.65 U/mL, respectively (Figure 3A). Compared to the CG group, DAO levels decreased by 35.50% in the LDP group and 28.57% in the HDP group, with both reductions being statistically significant (*p* < 0.0001). Similarly, D-LA concentrations declined from 0.44 mmol/L in the CG group to 0.35 mmol/L in the LDP group and further to 0.26 mmol/L in the HDP group, corresponding to reductions of 20.45 and 40.91%, respectively (Figure 3B). Statistical analysis confirmed that the decrease in D-LA levels was significant in the HDP group compared to both the CG (*p* < 0.001) and LDP groups (*p* < 0.05). LPS levels followed a similar trend (Figure 3C), with concentrations of 0.52 EU/mL in the CG group, 0.45 EU/mL in the LDP group, and 0.32 EU/mL in the HDP group, representing reductions of 13.46 and 38.46%, respectively. The HDP group exhibited a significant reduction in LPS levels compared to the CG (*p* < 0.001) and LDP groups (*p* < 0.01). These findings suggest that postbiotic treatment effectively reduces DAO, D-LA, and LPS levels in a dose-dependent manner, indicating a potential enhancement of gut barrier integrity.

### 3.5 Immunoglobulin levels

**Figure 4:**
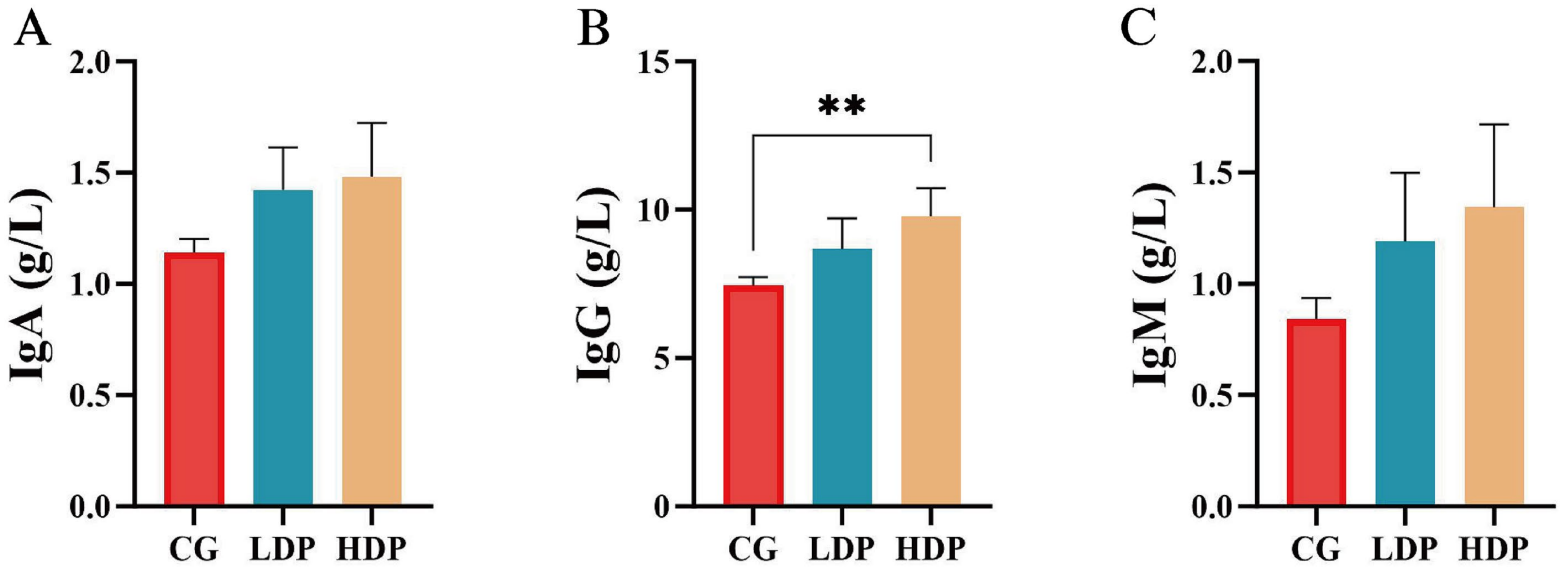
Effects of postbiotics on immunoglobulin parameters in cats. **(A)** IgA, immunoglobulin A, **(B)** IgG, immunoglobulin G, and **(C)** IgM, immunoglobulin M. Data are expressed as means ± SEM (*n* = 6). Statistical significance was analyzed using one-way ANOVA followed by Tukey’s post-hoc test. Significant differences are indicated as **, *p* ≤ 0.01.

The immunological response was evaluated by measuring IgG, IgM, and IgA concentrations, as illustrated in Figure 4. IgA (Figure 4A) and IgM (Figure 4C) levels did not show statistically significant differences among the CG, LDP, and HDP groups. However, IgG (Figure 4B) concentrations exhibited a notable upward trend, increasing from 7.44 g/L in the CG group to 8.67 g/L in the LDP group and 9.78 g/L in the HDP group, corresponding to an increase of 16.54% in the LDP group and 31.46% in the HDP group (*p* < 0.01) compared to the CG group.

### 3.6 Inflammatory cytokine response

**Figure 5:**
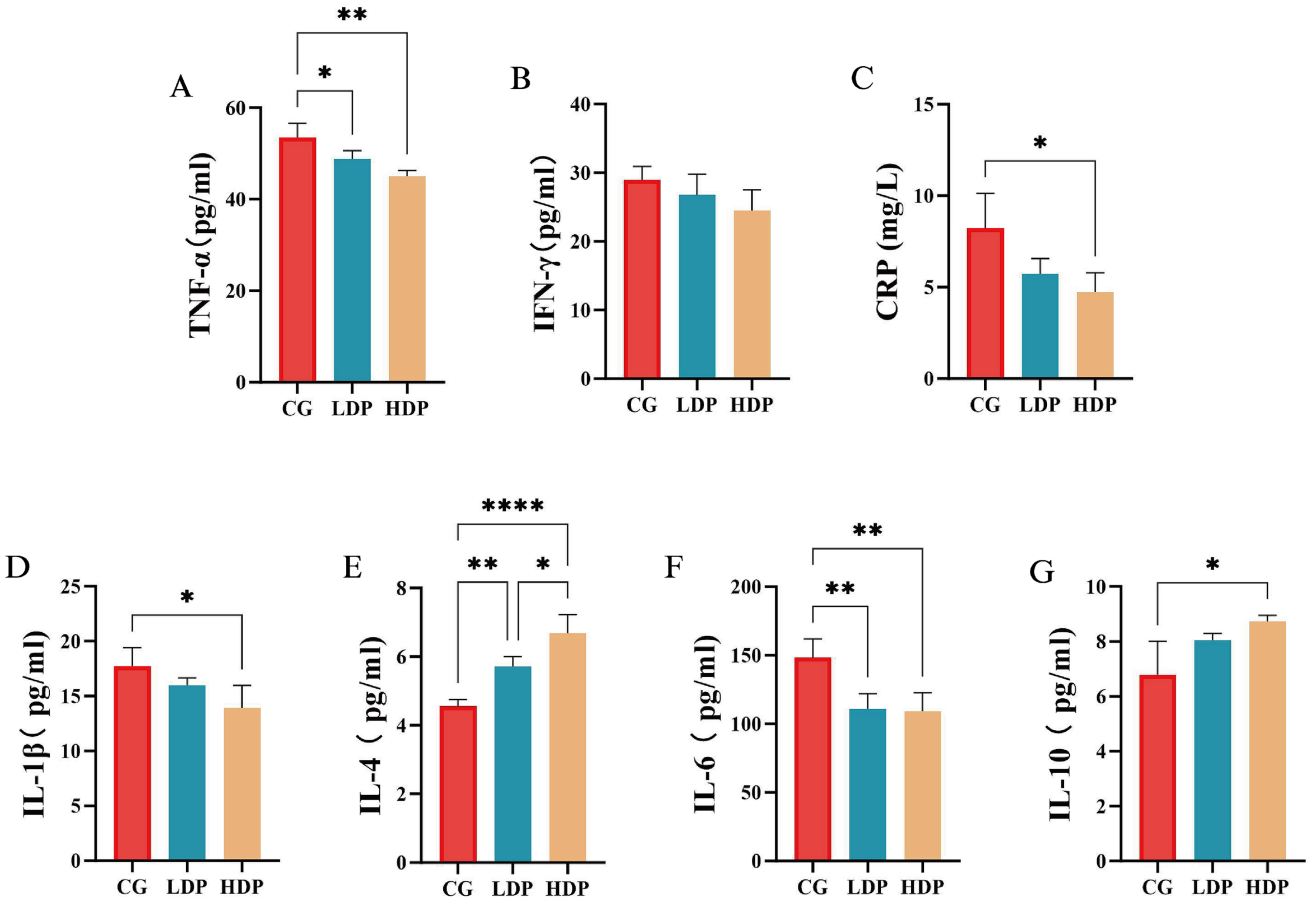
The effects of postbiotics on plasma inflammatory parameters in cats. **(A)** TNF-*α*, tumor necrosis factor-α, **(B)** IFN-*γ*, interferon-γ, **(C)**CRP, C-reactive protein, **(D)** IL-1β, Interleukin-1 beta, **(E)** IL-4, interleukin-4, **(F)** IL-6, interleukin-6 and **(G)** IL-10, interleukin-10. Data are expressed as means ± SEM (*n* = 6). Statistical significance was analyzed using one-way ANOVA followed by Tukey’s post-hoc test. Significant differences are indicated as * *p* ≤ 0.05**, *p* ≤ 0.01 and **** *p* ≤ 0.0001.

The concentrations of pro-inflammatory and anti-inflammatory cytokines were measured to assess immune regulation among the CG, LDP, and HDP groups following postbiotic treatment, as depicted in Figure 5. TNF-*α* levels (Figure 5A) exhibited a decreasing trend across the groups, with concentrations of 53.53 pg./mL in the CG group, 48.82 pg./mL in the LDP group, and 45.01 pg./mL in the HDP group. Compared to the CG group, TNF-α levels were reduced by 8.81% in the LDP group and 15.90% in the HDP group, with the latter showing a statistically significant decrease (*p* < 0.01), indicating a potential anti-inflammatory effect of postbiotic intervention. Similarly, CRP levels (Figure 5C) significantly declined following postbiotic administration. The CG group had a CRP concentration of 8.21 mg/L, which decreased to 5.72 mg/L in the LDP group (30.31% reduction, *p* < 0.05) and further to 4.73 mg/L in the HDP group (42.39% reduction, *p* < 0.05). The observed reductions suggest that postbiotic supplementation may help mitigate systemic inflammation. IL-1β levels (Figure 5D) followed a similar downward trend, with concentrations of 17.73 pg/mL in the CG group, 15.98 pg/mL in the LDP group, and 13.94 pg./mL in the HDP group. This corresponds to reductions of 9.87% in the LDP group and 21.38% in the HDP group, with the HDP group exhibiting a statistically significant decrease compared to the CG group (*p* < 0.05). IL-6 (Figure 5F) concentrations were also notably affected by postbiotic treatment, decreasing from 148.50 pg./mL in the CG group to 110.91 pg/mL in the LDP group (25.32% reduction, *p* < 0.01) and 109.31 pg/mL in the HDP group (26.41% reduction, *p* < 0.01). Both LDP and HDP groups exhibited significant reductions, suggesting that postbiotic administration effectively suppressed IL-6-mediated inflammation.

Conversely, IL-4 (Figure 5E) and IL-10 (Figure 5G) levels exhibited an increasing trend, suggesting an enhanced anti-inflammatory response. IL-4 concentrations were 4.56 pg/mL in the CG group, 5.71 pg./mL in the LDP group, and 6.68 pg/mL in the HDP group, reflecting an increase of 25.22% in the LDP group (*p* < 0.01) and 46.49% in the HDP group (*p* < 0.0001) compared to the CG group. Similarly, IL-10 levels increased from 6.78 pg/mL in the CG group to 8.06 pg/mL in the LDP group (18.88% increase, *p* < 0.05) and 8.74 pg/mL in the HDP group (28.91% increase, *p* < 0.05), indicating a significant upregulation in response to postbiotic treatment.

### 3.7 Fecal microbiota composition

**Figure 6:**
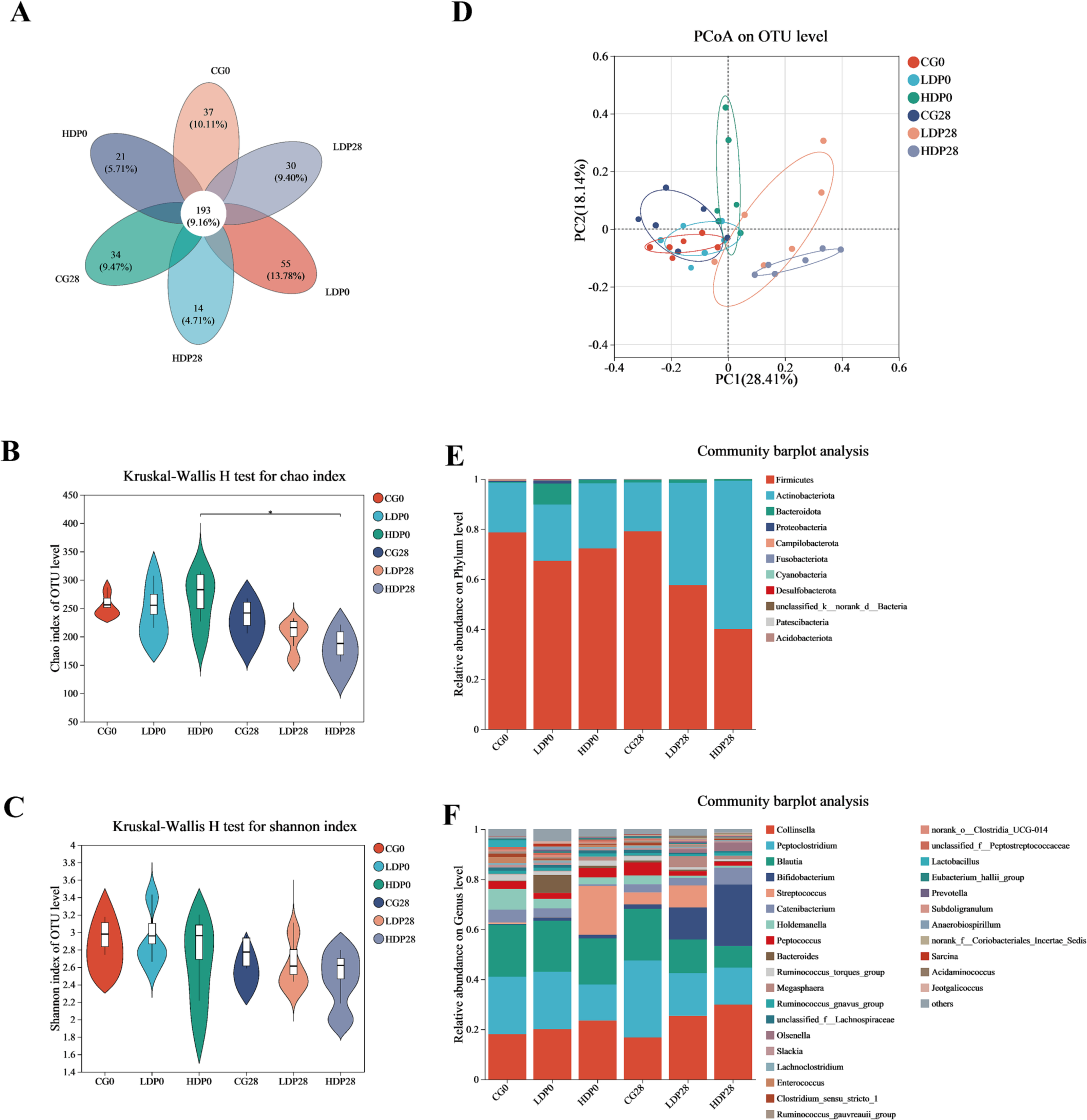
Effects of postbiotics on the composition of fecal microbiota in cats. **(A)** Venn; **(B)** Chao index; **(C)** Shannon index; **(D)** Principal Coordinates Analysis; **(E,F)** Phylum and genus level of bacterial, respectively. The values are expressed as means ± SEM, *n* = 6 per group.

As shown in Figure 6A, the Venn diagram illustrates the distribution of OTUs among the six groups, identifying a core microbiota composed of 193 shared OTUs across all groups. Each group also harbored unique OTUs, with the LDP0 group exhibiting the highest number and the HDP28 group the lowest. The Chao1 index results (Figure 6B) revealed a significant reduction in species richness within the HDP group after 28 days of postbiotic supplementation (*p* < 0.05), while no significant differences were detected among the other groups. In contrast, microbial diversity assessed by the Shannon index (Figure 6C) remained stable, showing no significant variation across groups (*p* > 0.05). Additionally, the PCoA plot (Figure 6D) displayed clear separation among microbial communities from different groups, particularly highlighting a notable shift in the HDP group from day 0 to day 28. The clustering of HDP28 samples indicates that postbiotic supplementation led to increased homogeneity in microbial community structure.

The variations in microbiota composition at the phylum level are illustrated in Figure 6E. Minimal shifts in microbiota composition were observed within the CG group. In contrast, substantial alterations occurred in both the LDP and HDP groups. Specifically, in the LDP group, the relative abundance of Firmicutes decreased from 67.3 to 57.5%, whereas Actinobacteriota increased from 22.4 to 40.9%. Notably, these changes were more pronounced in the HDP group, where Firmicutes substantially decreased from 79.1 to 40.1%, and Actinobacteriota markedly increased from 26.0 to 59.3%. At the genus level, as depicted in Figure 6F, the initial microbial composition of the CG0 group consisted primarily of *Collinsella* (18.0%), *Peptoclostridium* (23.0%), *Blautia* (20.8%), and *Bifidobacterium* (0.27%). After 28 days of feeding, the corresponding abundances in the CG28 group were slightly altered to 16.7, 30.8, 20.6, and 1.78%, respectively. In the LDP28 group, after the same duration, *Collinsella* decreased from 20.0 to 16.7%, *Peptoclostridium* from 22.9 to 17.0%, and *Blautia* from 18.5 to 13.4%; conversely, *Bifidobacterium* notably increased from 1.22 to 12.89%. Meanwhile, the HDP group exhibited significant elevations in the relative abundances of *Collinsella* and *Bifidobacterium* after 28 days; *Collinsella* increased from 23.5 to 29.8%, and *Bifidobacterium* demonstrated a substantial elevation from 1.46 to 24.6%.

**Figure 7:**
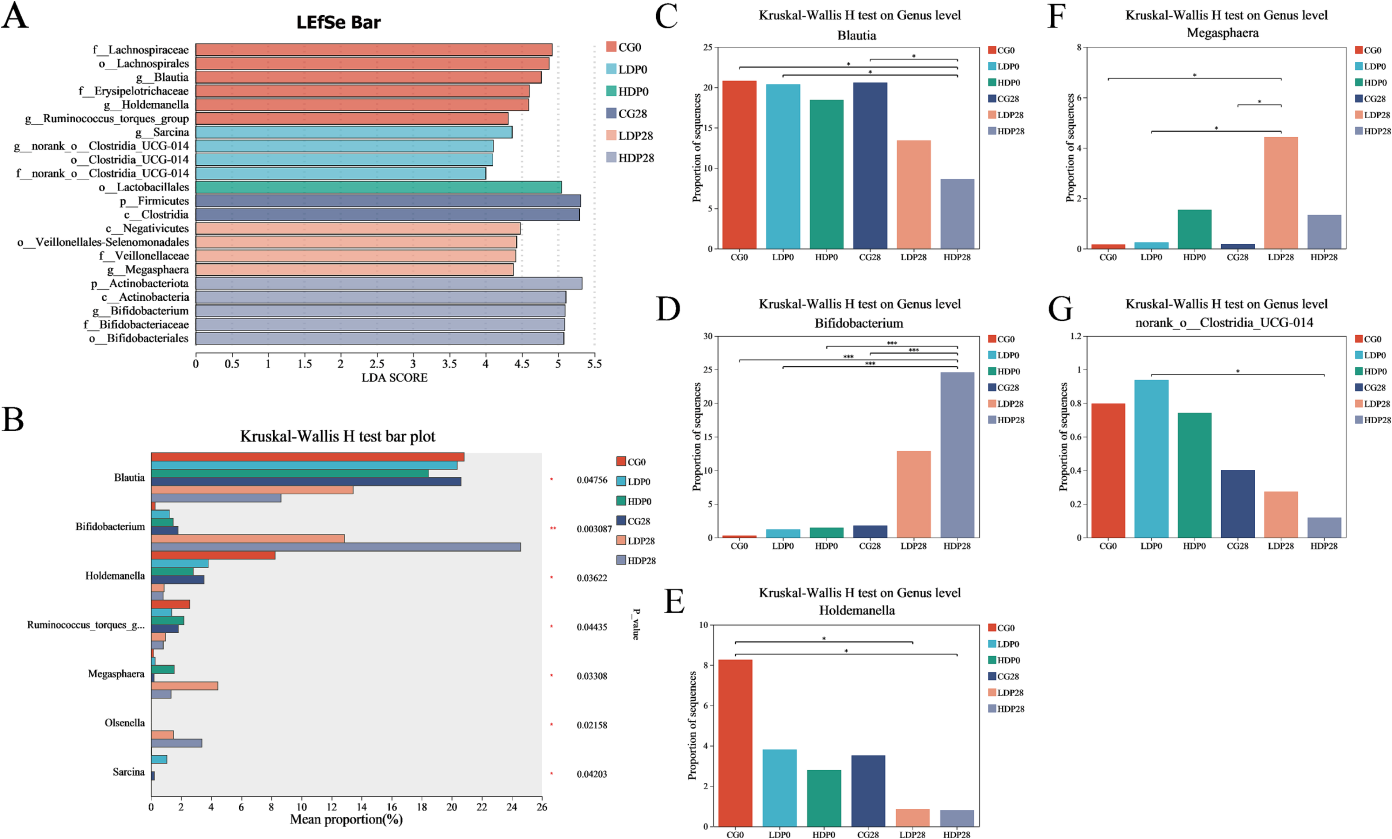
Effect of postbiotics supplementation on feline gut microbiota composition. **(A)** LEfSe (linear discriminant analysis effect size) bar plot; **(B)** Kruskal–Wallis H test bar plot; panels **(C–G)** depict the relative abundance of key microbial genera: **(C)** *Blautia*, **(D)** *Bifidobacterium*, **(E)** *Holdemanella*, **(F)** *Megasphaera* and **(G)** *norank_o_Clostridia_UCG-014*. Statistical significance: * *p* ≤ 0.05 and *** *p* ≤ 0.001. Data are represented as mean ± SEM based on a sample size of *n* = 6 per group.

Microbial composition analyses employing LEfSe (Linear discriminant analysis Effect Size) and Kruskal-Wallis H tests revealed pronounced and statistically significant shifts in genus-level microbiota across experimental groups (Figure 7). Specifically, LEfSe analysis (Figure 7A) pinpointed distinctive biomarkers, notably identifying significant enrichment of beneficial genera such as *Megasphaera* and *Bifidobacterium* within postbiotic -supplemented groups. In contrast, *Clostridia-*related genera were characteristically enriched within control groups, underscoring a postbiotic -driven selective microbiota modulation. Complementarily, the Kruskal-Wallis H test (Figure 7B) validated these genus-level microbial alterations, reinforcing the robustness of the observations regarding *Blautia*, *Bifidobacterium*, *Holdemanella*, and *Megasphaera*. *Blautia* exhibited a significant reduction in abundance within both LDP28 and HDP28 groups relative to CG (*p* < 0.05; Figure 7C). Conversely, the abundance of *Bifidobacterium* showed a pronounced dose-dependent enhancement, particularly emphasized in the HDP28 group when compared to all other treatment conditions (*p* < 0.001; Figure 7D). Notably, *Holdemanella* populations were significantly lower in both LDP28 and HDP28 groups compared with the control, suggesting a postbiotic-associated reduction (*p* < 0.05; Figure 7E). *Megasphaera* abundance was distinctly augmented in the LDP28 group, highlighting a unique postbiotic dose-specific microbial shift relative to all other groups (*p* < 0.05; Figure 7F). Furthermore, the abundance of *norank_o_Clostridia_UCG-014* significantly declined in postbiotic -supplemented conditions, with the greatest suppression observed in the HDP28 group compared to CG (Figure 7G).

### 3.8 Correlation between gut microbiota and serum indices

**Figure 8:**
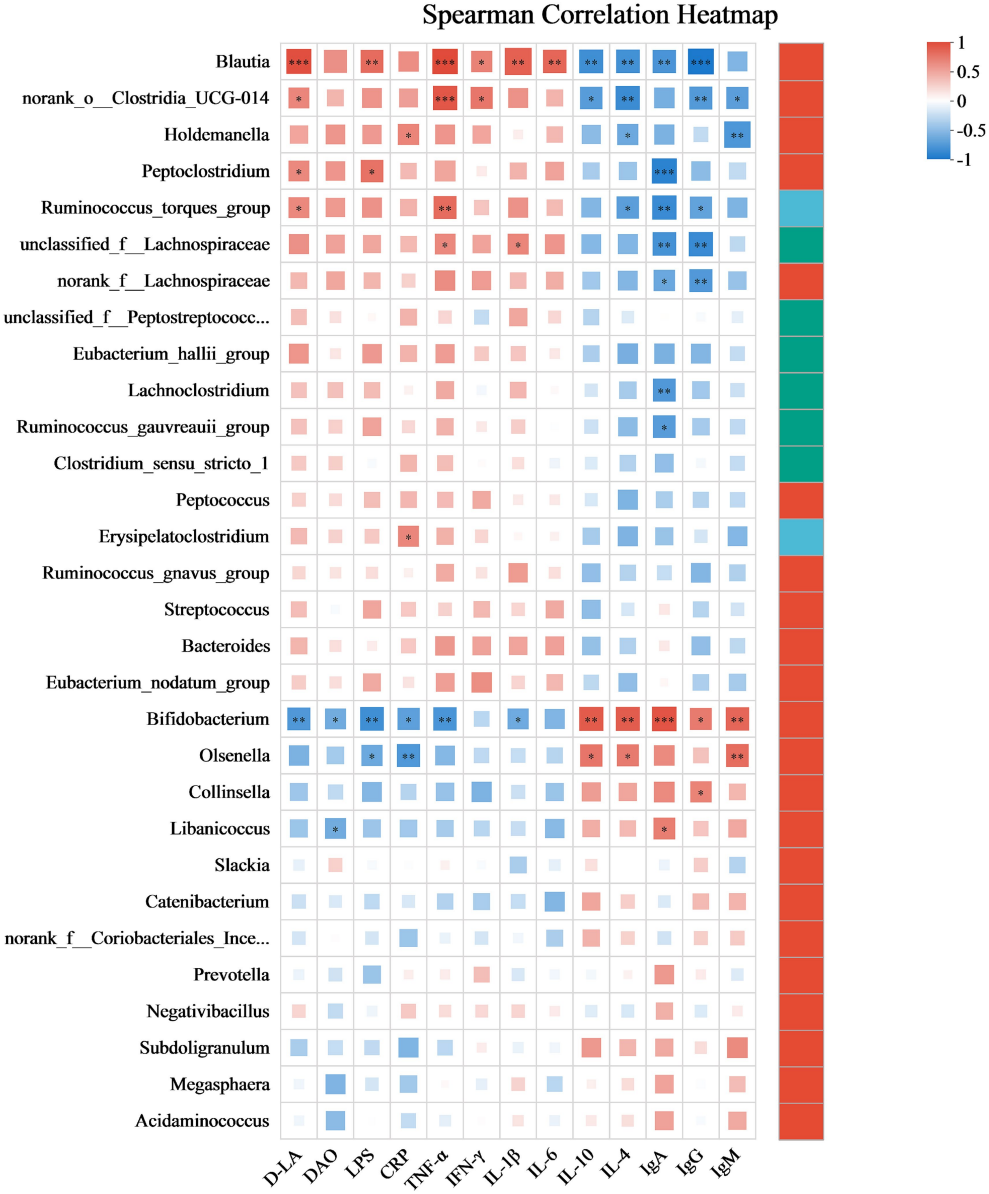
Spearman’s correlation heatmap depicting the associations between bacterial profiles and serum indices. Correlations are color-coded, with red representing positive associations and blue representing negative ones. The significance levels are * *p* ≤ 0.05, ** *p* ≤ 0.01, and *** *p* ≤ 0.001. After adjustment for multiple comparisons using the Benjamini–Hochberg false discovery rate (FDR) method. Data are presented as mean ± SEM, with a sample size of *n* = 6 per group.

This section elucidates the correlation patterns observed among the 30 most abundant bacterial taxa and various serum indices, as illustrated in Figure 8. *Blautia* exhibits a strong positive correlation with D-LA (*r* = 0.94), LPS (*r* = 0.73), TNF-*α* (*r* = 0.90), IFN-*γ* (*r* = 0.61), IL-1β (*r* = 0.78) and IL-6 (*r* = 0.76), but a significant negative correlation with IL-10 (*r* = −0.79), IL-4 (*r* = −0.78), IgA (*r* = −0.75) and IgG (*r* = −0.94). *norank_o__Clostridia_UCG-014* shows a positive correlation with D-LA (*r* = 0.59), TNF-α (*r* = 0.83) and IFN-γ (*r* = 0.67), but a negative correlation with IL-10 (*r* = −0.69), IL-4 (*r* = −0.81), IgG (*r* = −0.75) and IgM (*r* = −0.68). *Holdemanella* has positive correlations with CRP (*r* = 0.60), but negative correlations with IL-4 (*r* = −0.61) and IgM (*r* = −0.74). *Peptoclostridium* is positively correlated with D-LA (*r* = 0.58) and LPS (*r* = 0.69), but negative correlation with IgA (*r* = −0.87). *Bifidobacterium* has positive correlations with IL-10 (*r* = 0.81), IL-4 (*r* = 0.80), IgA (*r* = 0.85), IgG (*r* = 0.65) and IgM (*r* = 0.76), but negative correlation with D-LA (*r* = −0.73), DAO (*r* = −0.59), LPS (*r* = −0.79), CRP (*r* = −0.67), TNF-α (*r* = −0.75) and IL-1β (*r* = −0.60).

### 3.9 Postbiotic effects on fecal SCFAs

**Figure 9:**
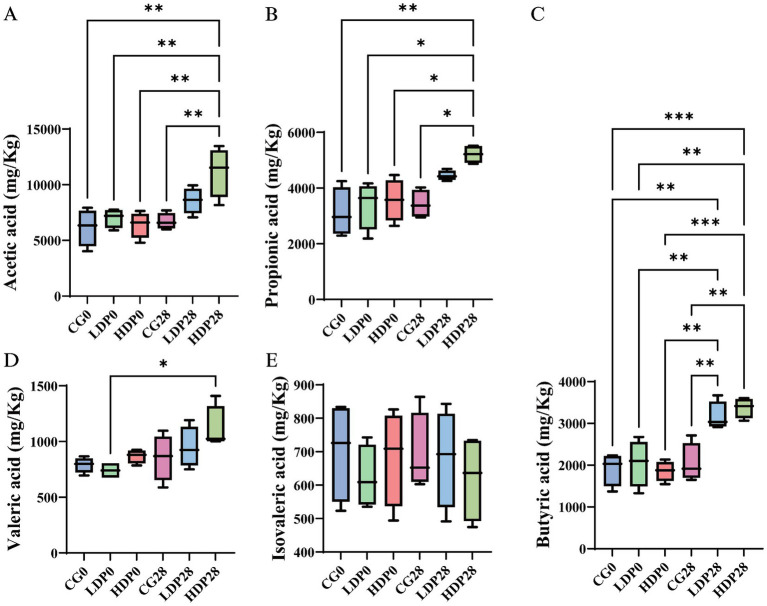
Effects of postbiotics on organic acid content in cat feces. **(A)** acetic acid, **(B)** propionic acid, **(C)** butyric acid, **(D)** valeric acid and **(E)** isovaleric acid. The significance levels are * *p* ≤ 0.05, ** *p* ≤ 0.01, and *** *p* ≤ 0.001. Data are presented as mean ± SEM, with a sample size of *n* = 6 per group.

The effects of postbiotics on the types and concentrations of organic acids in cat feces are illustrated in Figure 9. After 28 days of dietary supplementation with postbiotics, the HDP28 group exhibited the highest concentrations of acetic acid and propionic acid, measured at 11,172.37 mg/kg and 5,208.24 mg/kg, respectively. Compared to day 0, these levels represented significant increases of 74.17 and 46.05%, respectively, and were significantly higher than those observed in the CG0 and CG28 groups (*p* < 0.05; Figures 9A,B). As shown in Figure 9C, the concentration of butyric acid increased significantly by 54.19 and 81.52% in the LDP28 (3,162.69 mg/kg) and HDP28 (3,374.62 mg/kg) groups, respectively, compared with baseline (day 0). Both groups showed significantly higher levels than the CG0 and CG28 groups (*p* < 0.05). The valeric acid content, illustrated in Figure 9D, reached the highest value in the HDP28 group (1,115.17 mg/kg). In contrast, no significant differences in isovaleric acid concentrations were observed among any of the groups (Figure 9E). Furthermore, isobutyric acid and caproic acid were not detected in any of the tested groups.

## 4 Discussion

LDP and HDP supplementation improved fur scores over 28 days, indicating better skin and coat health. These findings align with previous research demonstrating the association between gut microbiota modulation and systemic health outcomes, including skin integrity and nutrient absorption (Prajapati et al., 2025). Notably, Body weight exhibited a consistent upward trend in all groups, and postbiotic intervention did not significantly alter this pattern, suggesting limited influence on metabolic balance or energy regulation. This observation supports earlier hypotheses proposing that modulation of the gut microbiota via probiotics or postbiotics can subtly influence metabolic pathways without disrupting energy balance (de Souza Junior and de Godoy, 2023). Additionally, fecal scores remained within the optimal range (2.5–3.0) in postbiotic-treated groups, further indicating improved gastrointestinal health. The underlying mechanisms likely involve favorable shifts in gut microbial composition, inhibition of pathogenic bacteria, and reinforcement of intestinal barrier function, consistent with findings reported in prior studies on companion animals (Opetz et al., 2023). Although no statistically significant differences were observed, WBC and HGB showed slight downward trends in postbiotic groups, while remaining within feline reference ranges. Similar findings have been reported with heat-treated *Bifidobacterium* preparations, which modulate inflammatory tone without causing hematological toxicity (de la Fuente-Muñoz et al., 2025). Recent feline studies also indicate that microbiota-targeted interventions can adjust immune markers without adverse effects on CBC (Wang et al., 2024).

Scanning electron microscopy showed smoother hair surfaces after postbiotic supplementation, with reduced scale thickness and shorter cuticle length. These structural refinements indicate enhanced hair integrity and align with previous reports linking gut microbiota modulation to improved skin and coat condition (Feng, 2024; Yang et al., 2023). While hair shaft diameter remained unchanged, the observed surface smoothness and reduced irregularities suggest that postbiotics exert their effects primarily on the hair’s outer morphology rather than core structure. Postbiotics may improve hair quality by modulating immunity, altering metabolism, and enhancing the follicular environment. They can shift T cell balance by suppressing Th17 responses and promoting regulatory T cells, which helps reduce local inflammation around hair follicles and supports a healthier growth environment (Kim et al., 2024). In parallel, postbiotic-derived SCFAs contribute to hair shaft integrity by promoting keratinocyte differentiation and reducing oxidative stress, both essential for maintaining smooth and structurally stable hair surfaces (De Almeida et al., 2023). Moreover, postbiotics may decrease the production of gut-derived toxins such as phenol and p-cresol, which are known to impair keratinocyte function and potentially disrupt hair shaft formation (Miyazaki et al., 2014). These combined actions suggest a mechanism through which postbiotics enhance hair surface architecture and shaft quality.

Lower serum DAO, D-LA, and LPS in postbiotic-treated cats indicate improved gut barrier function. As a marker of epithelial integrity, elevated DAO reflects mucosal damage and increased permeability; its decline suggests enhanced mucosal stability (Wang et al., 2024). Similarly, reduced D-LA levels imply diminished bacterial translocation and improved tight junction integrity (de Souza et al., 2022). Interestingly, DAO reduction was more pronounced in the low-dose postbiotic group than in the high-dose group. This non-linear effect has also been reported in other studies, where moderate doses of postbiotics improved barrier function effectively, but higher doses provided little added benefit (Cercamondi et al., 2025; Guo et al., 2025). This may reflect a saturation effect in host–microbe interactions or feedback regulation. Importantly, both doses in our study improved gut barrier integrity compared with control, supporting their practical value. The concurrent decrease in LPS, a key mediator of systemic inflammation and gut-derived endotoxemia, underscores the anti-inflammatory and microbiota-modulating properties of postbiotics, particularly in suppressing gram-negative bacterial activity (Algieri et al., 2023). These effects are consistent with postbiotics’ known mechanisms, including the generation of bioactive metabolites such as SCFAs, which promote epithelial repair and immune homeostasis (Martorell et al., 2021). Moreover, prior studies demonstrate that postbiotics sustain tight junction protein expression and downregulate pro-inflammatory cytokines, further contributing to intestinal barrier integrity (Vance et al., 2022). The reductions in DAO, D-LA, and LPS observed in this study indicate strengthened gut barrier integrity. Prior studies have shown that postbiotics can improve barrier function through upregulation of tight junction components, including ZO-1, occludin, and claudin (Zhang et al., 2022; Scott et al., 2022; Tong et al., 2025). Thus, our results are consistent with the hypothesis that postbiotics may exert barrier-protective effects by enhancing tight junction integrity, leading to reduced paracellular permeability and endotoxin translocation.

Postbiotic-induced enhancement of serum IgG levels suggests a stimulatory effect on humoral immune responses, likely driven by microbial metabolites such as SCFAs that promote B cell maturation and modulate cytokine-mediated signaling (Aguilar-Toalá et al., 2018). Postbiotics, consisting of inactivated microbial cells and their metabolites, engage gut-associated lymphoid tissue (GALT) to promote systemic immune responses (Tsilingiri and Rescigno, 2013). Evidence from both human and animal studies shows that postbiotics enhance immune homeostasis, suppress inflammatory markers, and increase antigen-specific IgG levels (Shenderov, 2013). In companion animals, microbiota-targeted interventions have been linked to improved immunoglobulin responses, supporting the relevance of postbiotics in feline immune modulation (Pilla and Suchodolski, 2020). These findings suggest that postbiotics can enhance systemic immunity without requiring live microbial colonization, offering a promising strategy for strengthening immune defenses in cats. In line with this, our study showed a clear dose–response increase in IgG following postbiotic supplementation, whereas IgA and IgM remained unchanged. In addition to systemic IgG, mucosal IgA plays a central role in gut immune defense. Although fecal IgA was not measured in this study, future investigations could address whether postbiotic supplementation also enhances mucosal IgA responses, thereby providing a more comprehensive view of humoral immune modulation. This indicates a selective humoral enhancement favoring systemic IgG, consistent with the notion that postbiotics fine-tune immune responses rather than broadly activating all antibody types (Asefa et al., 2025; Guo et al., 2025).

The observed reduction in pro-inflammatory cytokines (TNF-*α*, IL-1β, IL-6, and CRP) alongside an increase in anti-inflammatory markers (IL-4, IL-10) following postbiotic supplementation in cats highlights the immunomodulatory potential of postbiotics in mammalian systems. Postbiotics have been shown to attenuate systemic inflammation through modulation of gut-associated lymphoid tissue and immune signaling cascades (Bingöl et al., 2025). The marked suppression of TNF-α and CRP in the HDP group is consistent with findings in rodent models, where postbiotics downregulated NF-κB signaling and mitigated cytokine storms in inflammatory conditions such as colitis (Li et al., 2024b). The concurrent decreases in IL-1β and IL-6 suggest a broader dampening of innate immune activation, likely mediated by SCFAs and peptidoglycan derivatives that modulate macrophage polarization and inhibit Toll-like receptor pathways (Magrys and Pawlik, 2023). Simultaneously, the upregulation of IL-4 and IL-10 implies an active promotion of anti-inflammatory responses, potentially via regulatory T cell activation and enhanced secretion of immunosuppressive cytokines (Timlin et al., 2024). Comparable outcomes in poultry and canine models further reinforce the capacity of dietary postbiotics to restore immune balance by elevating IL-10 while reducing pro-inflammatory mediators (Danladi et al., 2024).

Postbiotic supplementation induced marked shifts in the feline gut microbiota, favoring the enrichment of beneficial genera such as *Bifidobacterium* and *Megasphaera*, which are known producers of SCFAs that support epithelial integrity and exert anti-inflammatory effects (Sharma et al., 2023; Beteri et al., 2024). Concurrent reductions in Firmicutes, *Blautia*, and *norank_o__Clostridia_UCG-014*—taxa often associated with dysbiosis, inflammation, and increased gut permeability—further underscore the microbiota-stabilizing effects of postbiotics (Bai et al., 2021; Motiani et al., 2020; Atuahene et al., 2024). Although microbial richness (Chao1 index) declined in the HDP28 group, overall diversity (Shannon index) remained stable, and PCoA revealed a clear compositional shift, suggesting enhanced ecological stability through selective modulation of bacterial communities (Wang et al., 2024; Zheng et al., 2021). These microbiota-level changes were dose-dependent, with *Bifidobacterium* showing the most prominent expansion, aligning with its recognized role in enhancing mucosal barrier function and systemic immune regulation (Favre et al., 2011). The enrichment of *Bifidobacterium* observed in the sequencing data may theoretically arise from residual DNA fragments of the heat-inactivated supplement or from stimulation of endogenous strains. Given the 28-day intervention and the metabolic instability of free bacterial DNA in the gut, the latter explanation appears more plausible, consistent with prior reports that postbiotic metabolites can promote the growth of beneficial commensals (Asefa et al., 2025; Guo et al., 2025). Nevertheless, future studies incorporating targeted qPCR or viability assays will be important to verify this interpretation.

Our findings are in line with recent feline-focused studies, which provide important species-specific context. Although most available feline studies have examined probiotics rather than postbiotics, their reported effects on gut microbiota and metabolites provide relevant parallels. Probiotic supplementation in cats with chronic kidney disease significantly enriched Actinobacteria and Firmicutes and improved metabolic profiles (Huang et al., 2025), paralleling the increase in SCFA-producing taxa observed in our trial. In kittens, a complex probiotic preparation modulated fecal metabolite profiles associated with inflammation and constipation, supporting the beneficial role of microbial interventions in feline gut health (Zhu et al., 2025). Moreover, alterations in microbiota composition and function, including reduced diversity and disrupted metabolic pathways, were summarized in cats with chronic enteropathies (Drut et al., 2024), findings that resonate with the microbial and metabolic differences we identified across intervention groups. A recent review also emphasized that cats and dogs exhibit distinct microbial and metabolic responses to dietary fibers and probiotics, underscoring the importance of feline-specific validation when interpreting our data (Lyu et al., 2025).

The correlations between specific bacterial taxa and serum biomarkers provide further mechanistic insight into the immunomodulatory effects of postbiotics. *Blautia* and *norank_o__Clostridia_UCG-014* exhibited strong positive associations with pro-inflammatory cytokines (TNF-*α*, IL-1β) and gut permeability markers (D-LA, LPS), alongside negative correlations with immunoglobulins and anti-inflammatory cytokines (IL-10, IL-4), indicating their potential involvement in systemic inflammation and barrier dysfunction. Conversely, *Bifidobacterium* positively correlated with IL-10 and IL-4, reinforcing its role in maintaining immune homeostasis and epithelial integrity (Yue et al., 2023; Mao et al., 2024; Dong et al., 2022). These patterns align with previous findings in feline models where heat-killed *Bifidobacterium animalis* supplementation attenuated inflammation and supported immune balance (de Souza et al., 2022). Moreover, *Peptoclostridium*’s positive correlation with D-LA and LPS implicates it in endotoxemia and intestinal permeability, consistent with its association with inflammatory enteropathies in cats (Yu et al., 2023). The strong correlations observed between *Blautia*, *Clostridia_UCG-014* and pro-inflammatory cytokines suggest that these taxa may serve as potential biomarkers of gut inflammation in cats, warranting further validation in disease models. Together, these results highlight the intricate interplay between microbiota composition and host immune status, and underscore the therapeutic potential of targeted postbiotic interventions in modulating gut-derived inflammation and promoting systemic immune resilience in companion animals.

Postbiotic supplementation significantly elevated fecal concentrations of major SCFAs: acetic, propionic, butyric, and valeric acids in cats, indicating enhanced microbial saccharolytic activity and improved colonic health. SCFAs are pivotal for gut homeostasis, reinforcing epithelial barrier integrity, regulating immune function, and serving as energy substrates for colonocytes (Liu et al., 2024). Notably, the marked increase in butyrate in the HDP28 group underscores its role in suppressing intestinal inflammation and promoting mucosal repair (Summers et al., 2020). The concurrent rise in valeric acid may further contribute to immune modulation and oxidative stress reduction, suggesting broader metabolic benefits (Oba et al., 2021). In contrast, the stable levels of isovaleric acid and the absence of isobutyric and caproic acids suggest a selective promotion of carbohydrate fermentation, without increasing protein-derived metabolites commonly associated with dysbiosis (Jewell and Jackson, 2020). Beyond local intestinal effects, SCFAs influence systemic immunity, lipid metabolism, and gut-brain axis signaling, reinforcing the therapeutic potential of postbiotics as a dietary strategy to optimize microbiota function and host immune balance (Rios-Covian et al., 2020). These findings warrant further investigation into their long-term physiological impacts and synergistic potential with other nutritional interventions.

## 5 Conclusion

This study underscores the potential of dietary postbiotics in promoting feline gastrointestinal and immune health. Cats receiving postbiotic supplementation exhibited notable improvements in gut barrier integrity, as reflected in the lowered levels of permeability biomarkers. Immune modulation was evident through elevated serum IgG and anti-inflammatory cytokines, while microbiota analysis revealed beneficial shifts in bacterial composition, including increased *Bifidobacterium* spp. and SCFA levels such as butyrate and acetate. These changes were accompanied by visible enhancements in fur condition, suggesting a broader systemic impact. Considering their heat stability and absence of risks tied to live organisms, postbiotics present a promising avenue for safe, long-term nutritional interventions in feline diets. Moving forward, it would be valuable to assess their efficacy in cats with preexisting gastrointestinal or immune disorders, and to explore their mechanisms of action at a molecular level.

## Data Availability

The datasets presented in this study can be found in online repositories. The names of the repository/repositories and accession number(s) can be found at: https://www.ncbi.nlm.nih.gov/, PRJNA1310671.

## Glossary

SCFAs: short-chain fatty acids
DAO: diamine oxidase
LPS: lipopolysaccharide
CG: control group
LDP: low-dose postbiotic group
HDP: high-dose postbiotic group
CBC: complete blood count
D-LA: D-lactate
ELISA: enzyme-linked immunosorbent assay
TNF-*α*: tumor necrosis factor-alpha
IFN-*γ*: interferon-gamma
CRP: C-reactive protein
IL-1β: interleukin-1 beta
IL-4: interleukin-4
IL-6: interleukin-6
IL-10: interleukin-10
IgA: Immunoglobulin A
IgG: Immunoglobulin G
IgM: Immunoglobulin M
PCoA: principal coordinate analysis
ANOVA: analysis of variance
GALT: gut-associated lymphoid tissue
